# Highly Efficient Nanostructured Bi_2_WO_6_ Thin Film Electrodes for Photoelectrochemical and Environment Remediation

**DOI:** 10.3390/nano9050755

**Published:** 2019-05-17

**Authors:** Bandar Y. Alfaifi, Hossein Bayahia, Asif Ali. Tahir

**Affiliations:** 1 Environment and Sustainability Institute (ESI), University of Exeter, Penryn Campus, Penryn, Cornwall TR10 9FE, UK; ba283@exeter.ac.uk; 2 Chemistry Department, Faculty of Science, Albaha University, Albaha 65527, Saudi Arabia; hbayahia@bu.edu.sa

**Keywords:** Bi_2_WO_6_, nanostructures, microsphere structures, thin films, photoelectrochemical, photocatalyst, methylene blue, rhodamine B

## Abstract

Nanostructured Bi_2_WO_6_ thin film electrodes with enhanced solar energy conversion and photocatalytic properties have been fabricated using Aerosol-Assisted Chemical Vapor Deposition (AACVD). By conveniently controlling the deposition process parameters, Bi_2_WO_6_ electrodes were fabricated with nanoplates and hierarchical buckyball-shaped microsphere structures morphology. A detailed study has been conducted to correlate the structure and morphology with the photoelectrochemical (PEC) and photocatalytic dye degradation performance. The PEC investigations revealed that the hierarchical buckyball-shaped microsphere structured Bi_2_WO_6_ electrodes have shown the photocurrent density of 220 μAcm^−2^ while nanoplates have a photocurrent density of 170 μAcm^−2^ at 0.23 V (vs. Ag/AgCl/3M KCl) under AM1.5 illumination. The PEC characterization of Bi_2_WO_6_ electrodes also reveals that the photocurrent density and photocurrent onset potential is strongly dependent on the orientation and morphology, hence the deposition parameters. Similarly, the methylene blue (MB) and rhodamine B (RhB) photodegradation performance of Bi_2_WO_6_ electrodes also show a strong correlation with morphology. This finding provides an appropriate route to engineer the energetic and interfacial properties of Bi_2_WO_6_ electrode to enhance solar energy conversion and the photocatalytic performance of semiconductor materials.

## 1. Introduction

The fabrication of nanostructured materials with well-defined shapes and morphology has been considered a great challenge in material chemistry and nanotechnology [1,2]. In recent times, the strong relationship between morphology and performance has been acknowledged as an important factor to be aware of while designing the profile and organization of nanomaterials [3,4]. Nanomaterials with well-designed surface features have been inspiring significant research due to their vital functions in the study of structure-property interactions [5,6]. Well-organized hierarchical morphologies of inorganic compounds offer innovative chemical, as well as physical, properties because the benefits of materials lie in the nature of their microstructure as well as their nanostructure [7]. The conventional procedures for scheming the morphology (structural arrangement and size) of nanomaterials are mainly dependent on two basic approaches: first is the chemical approach which includes the hydrothermal process [8], surfactant templating [9], as well as molecular assembly [10] and so on; the second is the physical strategy, mainly based on thermal evaporation [11] and physical exfoliation [12].

Despite the successful preparation of monodispersed spherical particles, one-dimensional nanorods and nanowires, the deposition of nanoelectrodes possessing the desired characteristics remains difficult. Several deposition procedures—viz. pulsed laser deposition [13], reactive sputtering [14], spin-coating [15], spray pyrolysis [16] sol-gel [17,18] and chemical vapor deposition [19]—have already been applied for the deposition of thin film electrodes with controlled shape and morphology.

Among the aforementioned electrode deposition methods, the aerosol-assisted chemical vapor deposition (AACVD) is facile, possesses advantages such as solo solution and may be applied to the preparation of multicomponent electrodes. Significant efforts have been made to prepare Bi_2_WO_6_ particles by hydrothermal process and then the deposition of thin film electrodes using the dip-coating method [20], spin coating [21], spray pyrolysis [22] and an electrostatic layer by layer deposition procedure [23]. However, to the best of author’s knowledge, some information is available in the literature regarding the deposition of Bi_2_WO_6_ electrodes using AACVD. Ishikawa et al. prepared Bi_2_WO_6_ thin films by metal organic chemical vapor deposition (MOCVD) utilizing a vertical cold-wall reactor. Trimethyl bismuth and pentaethoxy tungsten have been applied as the Bi and W precursors in the presence of O_2_ gas as an oxidant [19].

Bi_2_WO_6_ has received noteworthy consideration owing to its successful use both as a photocatalyst [24] and a semiconductor material in photoelectrochemical (PEC) water splitting [25,26,27,28,29] as well as in CO_2_ reduction [30]. It has been reported that the photocatalytic activity and PEC characteristics of Bi_2_WO_6_ nanomaterials closely correlate with their particle dimensions and surface features, as well as their structural organization.

In most photocatalytic processes, a suspended powder is used as a photocatalyst with reduced photocatalytic activity. A difficult recollection process is a major barrier to realizing a large scale application of photocatalysis [31]. Nanostructured thin film electrode has the potential to overcome this barrier and nanostructured electrodes possess many advantages such as a high internal surface aspect ratio, excellent porosity and tremendous photocatalytic activity [31]. However, the problem of synthesizing cost-effective crystalline nanofilm electrodes of Bi_2_WO_6_ on a transparent substrate has not yet been fully resolved.

In this work, Bi_2_WO_6_ nanostructured electrodes with different morphologies were fabricated by Aerosol Assisted Chemical Vapor Deposition (AACVD). The cost-effective and facile AACVD approach is reproducible. We have fabricated Bi_2_WO_6_ nanostructured electrodes that have diverse morphologies, for instance nanoplates and hierarchical buckyball-shaped microsphere structures, by expediently controlling the process factors of AACVD. Moreover, the electrodes prepared in this work have shown remarkable photoelectrochemical activity and efficient photocatalytic degradation of methylene blue (MB) and rhodamine B (RhB) dyes. Our research will certainly contribute considerably to the future development of Bi_2_WO_6_ as a prospective semiconductor material for related such as electrochemical energy storage, photocatalysis, self-cleaning, sensors, photo-responsive switches, thermoelectric devices and electro-ceramics. The preparation method described here has the ability to expand the morphology controlled fabrication of related materials.

## 2. Experimental

### 2.1. Materials

Fluorine-doped tin oxide (FTO) coated glass (TEC 8 Pilkington, 8 Ω/square) were employed as the substrate and were cleaned by sonication in acetone, ethanol and distilled water for 10 min each and stored in ethanol until later use. All other analytical grade chemicals and reagents were purchased from Sigma-Aldrich (UK) and used without further purification. Distilled water was used throughout the experiment.

### 2.2. Preparation of Precursor Solution

0.9 g of Bi_2_O_3_ powder and 0.5 g of (NH_4_)_6_ H_2_W_12_O_40_·XH_2_O powder were added to a mixture of 10 mL of acetic acid and 5 mL of dimethylaminopropanol (DMAP). The above suspension was stirred and heated to 100 °C until the mixture became a transparent solution. Once the above solution became clear and after cooling to room temperature, the mixture was diluted to 100 mL by adding dimethylformamide to make the precursor solution.

### 2.3. Fabrication of Electrodes by AACVD

In a typical deposition, 15 mL precursor solution was taken in a 50 mL round-bottom flask and placed above the piezoelectric modulator of an ultrasonic humidifier. Air at a flow rate of 300 mL/min was used as the carrier gas and the flow rate was controlled by a L1X linear flowmeter. The generated aerosol droplets of the precursor were transferred to the heated reactor by the carrier gas [32]. The substrate was placed in the reactor (hotplate surface) so that aerosol reaches the Fluorine doped tin oxide (FTO) glass substrate and falls vertically from the top. The substrate was heated up to the deposition temperature for 20 min before the deposition starts. The deposition experiments were repeated several times to verify the reproducibility and controllability of the process. Controlled experiments were also carried out using an aqueous solution of Bi(NO_3_)_3_·5H_2_O and (NH_4_)_6_ H_2_W_12_O_40_·XH_2_O.

### 2.4. Material Characterization

The material phase composition was determined using a Bruker D8 Advance X-ray diffractometer (XRD, Billerica, MA, USA) (Cu Kα irradiation, 40 kV/40 mA, 0.02° 2θ step size and a scan time of 3 s per step) in the range of 20–70° 2θ. The morphology and composition of the thin film were characterized using a high-resolution scanning electron microscope (HITACHI S3200N SEM, Tokyo, Japan) coupled with energy dispersive spectroscopy (EDS; Oxford instrument elemental analysis). Raman spectroscopy was undertaken using a HORIBA Jobin Yvon LabRAM HR Raman Spectrophotometer, Kyoto, Japan (with 632.8 nm He-Ne laser). The spectrum was recorded in the range of 100 to 1600 cm^−1^.

Absorption spectra were measured on a lambda 1050 with 150 mm integrated InGaAs sphere Perkin-Elmer UV-Vis spectrophotometer in the wavelength range of 200–800 nm.

### 2.5. Photoelectrochemical and Photocatalytic Characterisation

The photoelectrochemical (PEC) efficiency of Bi_2_WO_6_ photoelectrodes was determined in 1 M NaOH solution (pH = 13), with the help of 300 W xenon lamp coupled with an AM 1.5 filter (Newport 66902). An electrochemical cell with three probes has been employed in the electrochemical observation. Linear sweep voltameter was employed for photocurrent calculation in +0.35 to −0.35 V range. Photodegradation of methylene blue (MB) and rhodamine B (RhB) was in 60 mL of a 10 mg/L solution using 1 mL of H_2_O_2_ as a catalyst. The degradation efficiency (C/C_0_), was calculated by measuring the dye absorption at wavelengths 653 nm and 554 nm for MB and RhB, respectively. Herein, the C_0_ indicates the initial concentration of the dye whereas C is the concentration at different time intervals.

## 3. Result and Discussion

In the present study, the synthesis of Bi_2_WO_6_ nanoelectrodes was accomplished using the one-step approach, utilizing AACVD procedures. As described in the experimental section of this manuscript, the impact of deposition temperature and carrier gas flow on the structure and photocatalytic activity of the electrodes was studied. Briefly, the electrodes deposition showed better performance compared to that in the existing literature, owing to one-step fabrication and deposition. The electrode showed fine adhesion and excellent stability during the photocatalytic experiments.

### 3.1. Material Characterization

**Figure 1:**
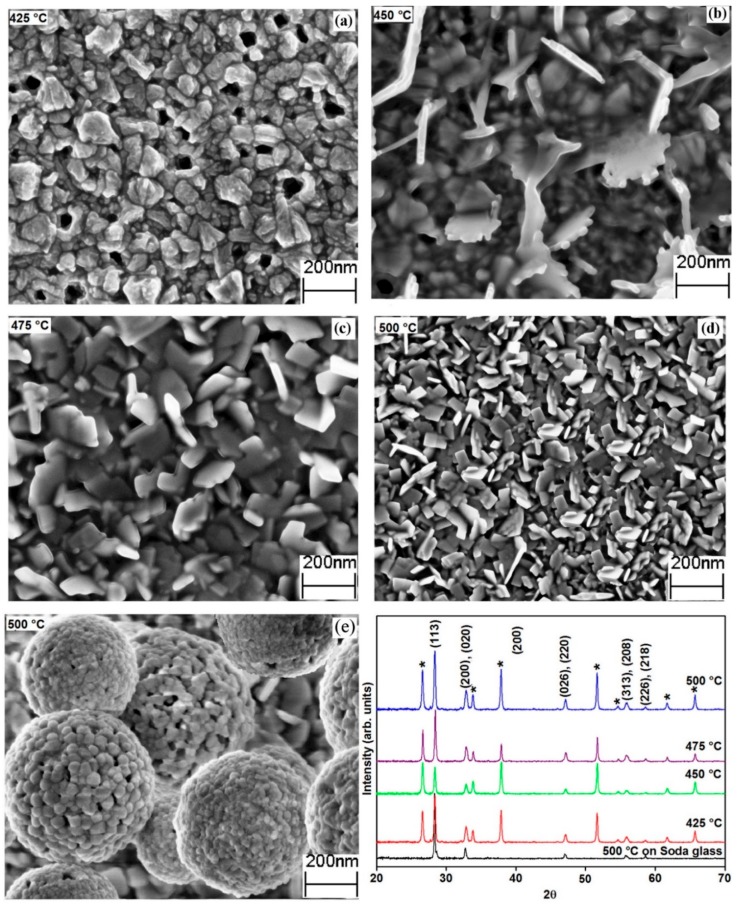
The scanning electron microscope (SEM) images (**a**–**d**) deposited at 300mL/mint flow rate (**e**) at 500 °C with 100 mL/min carrier gas flow. The X-ray diffraction (XRD) spectra of electrodes at deposition temperature of 425–500 °C, the peaks marked with (*) belong SnO_2_ arises from the Fluorine doped tin oxide (FTO) substrate.

The XRD spectra of the deposited electrodes correspond to the orthorhombic phase (Russellite) Bi_2_WO_6_ with space group B2ab (Figure 1), in addition to the peaks indexed as Fluorinated SnO_2_ layer used as substrate. The obtained XRD spectra are consistent with previous spectra present in the literature for Bi_2_WO_6_ (ICDD 01-073-2020) [33].

The Raman spectroscopic data of Bi_2_WO_6_ electrodes is illustrated in Figure S1 (supporting information) and the peaks at 760 and 790 cm^−1^ were indexed to the asymmetric and symmetric A_g_ modes of terminal O–W–O chain in Bi_2_WO_6_ [34]. The peak at 310 cm^−1^ was assigned to Bi^3+^ and WO_4_ whereas the terminal WO_2_ group was assigned to peaks at 300 cm^−1^. The peaks in the wavelength range of 700–1000 cm^−1^ were assigned to W–O bonds [35].

### 3.2. Deposition Temperature Effect on Morphology and Crystallinity

The impact of deposition condition on the morphology of Bi_2_WO_6_ electrodes was carried out extensively in the present study. Different substrate temperatures were applied to find out the effect on the crystallinity and morphology, however; the rest of the variables remained the same.

To investigate the deposition temperature effect on structure and morphology, at various temperatures, electrodes were deposited by using AACVD. Figure 1 depicts the XRD patterns of Bi_2_WO_6_ electrodes at various deposited temperatures with their respective morphology and structure. The XRD peaks were prominent and intensify gradually, indicating better crystallinity with increasing temperatures (425–500 °C). The unidentified peaks present in the spectra of XRD (Figure 1) of the electrode deposited at 425 °C disappeared with the rise in temperature, signifying that these peaks may correspond to un-decomposed materials. The crystallite size calculated on the basis of peak expansion (113) using the Scherrer equation is about 41 nm. The SEM micrograph of the electrode (Figure 1a) indicates a dense homogeneously dispersed microstructure without any distinguishable features. As shown in Figure 1b, nanoplates started to emerge from the dense microstructures, as the temperature of the deposition increased to 450 °C. Figure 1b illustrates that the size and form of the nanoplates are not well defined although long edges were clearly visible. As the temperature of deposition was further amplified to 475 °C, the electrode structure depicted specific plate-like morphology possessing narrow edges. An additional increase in temperature of deposition to 500 °C resulted in big and thin nanoplates. The length of the edge of an average nanoplate is about 110 nm, with a width of about 10 nm. Figure 1d demonstrates that the nanoplates that emerged from the substrate were aligned perpendicular to the FTO base.

### 3.3. Effect of Carrier Gas on Morphology

**Figure 2:**
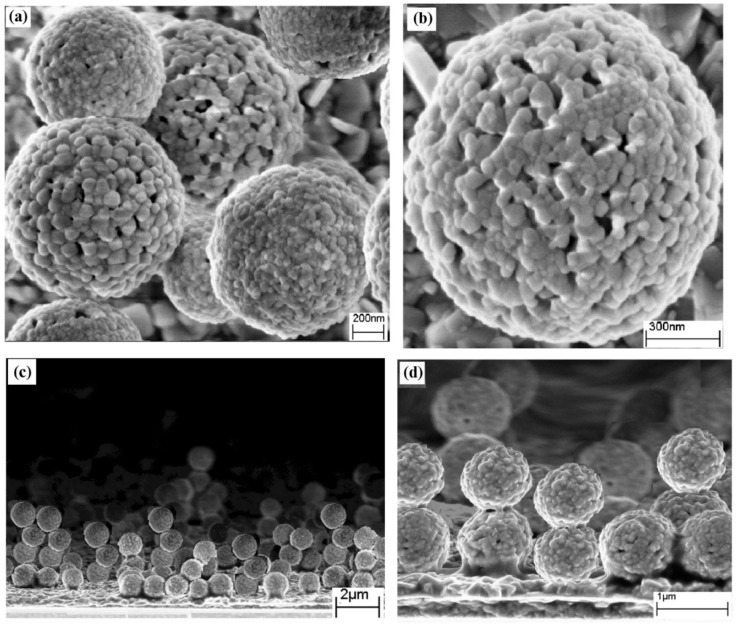
The SEM micrographs of hierarchical buckyball-shaped microsphere structured electrodes deposited by Aerosol-Assisted Chemical Vapor Deposition (AACVD) at 500 °C: (**a**,**b**) surface morphology, (**c**,**d**) cross section of electrodes.

Although the carrier gas flow can potentially influence the deposition temperature due to the cooling effect, the change of carrier gas flow had no pronounced effect on the morphology of electrodes deposited below 500 °C. Surprisingly, the amplification of the temperature of the deposition to 500 °C and simultaneous reduction of the carrier gas flow rate to 100 mL/min resulted in the formation of hierarchical buckyball-shaped microsphere structured electrodes (Figure 1e). Further SEM analysis (Figure 2a,c) showed that the buckyball-shaped microsphere structure covers almost the entire FTO surface, constructing a very uniform electrode of Bi_2_WO_6_. The high-resolution image of each sphere (Figure 2b) revealed that Bi_2_WO_6_ have superstructures with a buckyball-shaped appearance. These microspheres were in the size range of 0.4 to 0.8 µm. It was understood that the superstructures are mainly composed of 2D nanoparticles with a width around 10–30 nm, as revealed by the SEM. The surfaces of the microspheres were coarse and had many individual nanoparticles. The SEM images also reveal that these nanoparticles were aligned perpendicular to the sphere, pointed to the shared core within the buckyball-shaped microstructure (Figure 2b). More importantly, the microspheres have numerous pores possessing diverse diameters, indicating the potential to provide improved physicochemical characters. Within the microspheres one can easily see some nanoplates formed on the surface of the FTO (Figure 2a,b), which suggests a two-stage growth process of the Bi_2_WO_6_ microspheres. The primary stage is the formation of nanospheres/nanoplates and then these nanoplates self-assembled to form microsphere structures, which is a common phenomenon in the formation of microspheres in the hydrothermal process [5,33]. A cross-section of the electrode (Figure 2c,d) showed the growth of the Bi_2_WO_6_ hierarchical structures on top of each other, increasing the thickness of the electrode up to 3 µm. The cross-section image further confirmed that each microsphere is about 0.4–0.8 µm in diameter.

The formation of nanoplates and microsphere structured-based electrodes was also evident from the analysis of XRD data. The intensity ratio of (113) peak to the (200) peak *I*_(113)_/*I*_(200)_ for the films deposited at 475 °C and 500 °C is 3.6 and 2.8, respectively, which are much smaller than the standard ratio value of five (ICDD 01-073-2020). This relatively low *I*_(113)_/*I*_(200)_ ratio indicates specific anisotropic growth of crystals along the plane (001). Further analysis shows that at low temperatures (i.e., 400 °C) the value of the intensity ratio of (113) peak to the (200) peak was near to the standard ratio of 5 and steadily declined with increasing temperature. These changes imply the anisotropic nature of the electrodes. This can be ascribed to their surface structures, which were further confirmed by SEM and agree well with the reported ratio of *I*_(113)_/*I*_(200)_ for the square plates like morphologies of Bi_2_WO_6_ [10].

### 3.4. Mechanism of Formation of Nanoplates and Growth of Hierarchical Superstructures by AACVD

**Figure 3:**
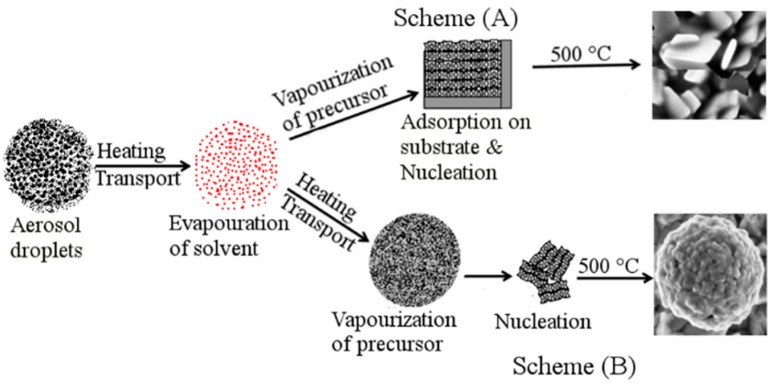
A diagrammatic representation of probable development process of nanoplate and hierarchical buckyball-shaped microsphere structures formation during AACVD process.

The formation of Bi_2_WO_6_ nanoplates and hierarchical microsphere structures by the hydrothermal process has been already explained based on the controlled concentration of Bi^3+^ ions by using organic chelating ligands or by increasing the pH of the solution [5,36]. Similarly, in our work, acetate and DAMP play a multifold role in the growth of Bi_2_WO_6_ nanoplates and hierarchical buckyball-shaped microsphere structures. Acetate acted as complexing agents in the precursor solution as it influenced nucleation and growth rate of Bi_2_WO_6_. For example, the carboxylate groups of acetate complex with bismuth ions decrease the free ion concentration in the solution. During the deposition process, aerosol droplets travel through different temperature zones; the first step is the evaporation of solvents and the second step is vaporization of the precursor. Therefore, when the acetate complex chelation becomes weak, the release of ions will take place. At a temperature of 450 °C, the released ions will be deposited on the substrate surface and subsequently, the nucleation starts. So, the nucleation and growth of particles will undergo a lengthy procedure and consequently results in uniform crystals. The carboxylate groups released are then adsorbed on the surfaces of the newly produced nanocrystals and facilitate the self-assembly to form nanoplates (scheme A in Figure 3).

On the other hand, the formation of hierarchical microsphere structures at an AACVD deposition temperature of 500 °C, using a precursor solution, might be defined as a homogeneous decomposition reaction. At a relatively high deposition temperature (i.e., 500 °C), the main decomposition process takes place during the vapor phase, therefore resulting in homogeneous nucleation which produces fine particles. These fine particles aggregate to form nanoparticles before they are adsorbed onto the substrate. The continuous flow of gases may help these nanoparticles to spin along the c-axis to build hierarchical microsphere structures (scheme B in Figure 3). The formation of hierarchical microsphere structures over the top of each other (Figure 2d) is clear evidence for the nucleation and formation of nanoparticles in the vapor phase before they plunge on to the substrate. The deposition process in AACVD is much more complex than the traditional CVD method [37]. Nevertheless, to date there is no specific model to portray the AACVD-procedures fully [38]. It has been hypothesized that the surfaces of nanobuilding blocks modified by organic surface groups form specific aggregation, whereas loosely arranged nanoparticles repeatedly lead to unsystematic aggregation [39]. The control experiments conducted using Bi(NO_3_)_3_·5H_2_O and (NH_4_)_6_ H_2_W_12_O_40_·XH_2_O solution in distilled water showed that the particles formed are irregular and dispersed randomly without dimethylaminopropanol (DMAP) ligands (Figure S2 in supporting information).

### 3.5. Optical Characterization

**Figure 4:**
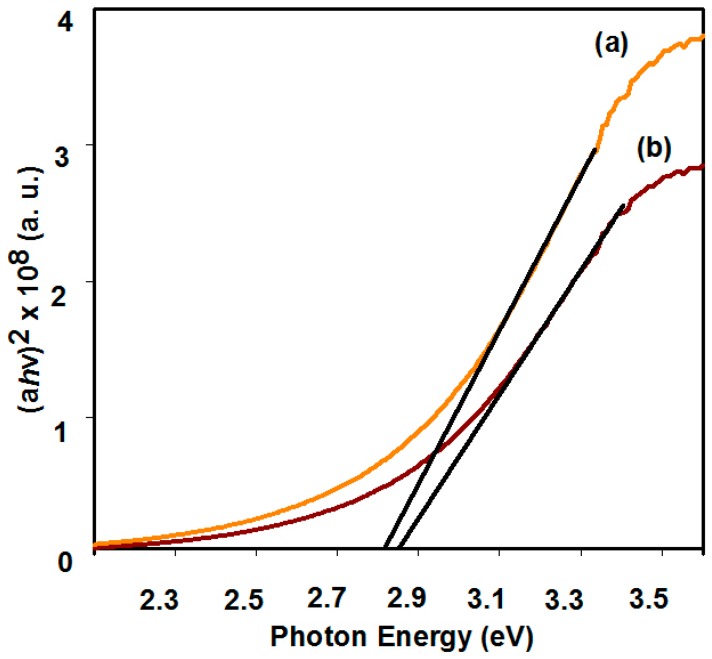
Plot of (*αh*υ)^2^ versus photon energy (*h*υ) for Bi_2_WO_6_ electrodes deposited by AACVD deposited at 500 °C, (**a**) hierarchical buckyball-like microsphere and (**b**) nanoplates.

The Bi_2_WO_6_ thin films possess sharp absorption peaks the in visible region [40]. The UV-visible spectra of Bi_2_WO_6_ electrodes have been taken by UV/Vis spectrophotometer. Figure 4 shows the bandgap of Bi_2_WO_6_ film electrodes, (a) for hierarchical buckyball-shaped microsphere structured electrodes deposited by AACVD at 500 °C and (b) for nanoplate-like electrodes deposited by AACVD at 500 °C. The bandgap (*E_g_*) of the electrodes was calculated using the Tauc plot equation (*αh*ν)^n^ = *A*(*h*ν − *E*_g_)and were found to be 2.81 and 2.85 eV, respectively. The results obtained are similar to those previously reported for Bi_2_WO_6_ powder nanoparticles [5].

### 3.6. Photoelectrochemical Characterisation

**Figure 5:**
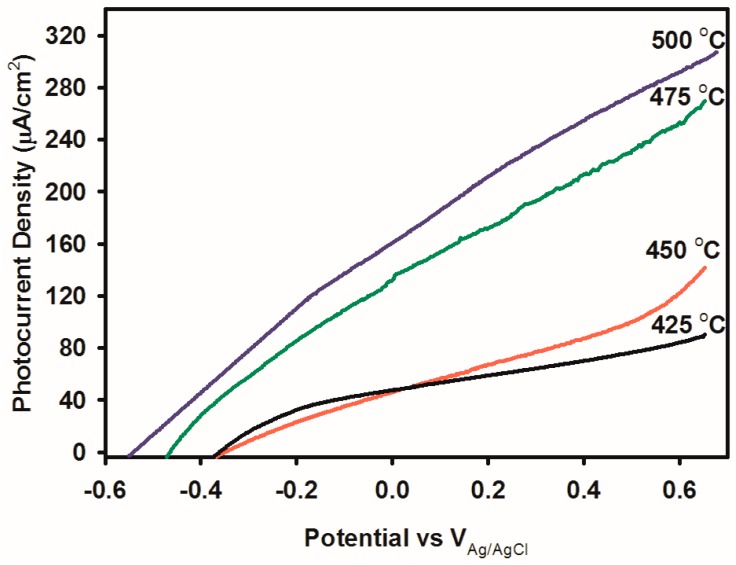
Photocurrent-voltage plots showing the deposition temperature dependence of photocurrent of Bi_2_WO_6_ electrodes deposited by AACVD.

The PEC measurements of the Bi_2_WO_6_ electrodes were studied by way of the 3-electrode approach, using AM 1.5 illumination. The current density plots in Figure 5 revealed the relation between photocurrent density and deposition temperature. The *J*-V curves in Figure 5 illustrate the change of photocurrent onset in the direction of negative potentials for hierarchical buckyball-shaped microsphere electrodes deposited by AACVD. The negative shift of the photocurrent may be as a result of (001) preferred plane orientation of the grain particles and electrodes’ surface structure. Hence, the data obtained confirm that there is a direct relationship between photocurrent and the deposition temperature. The grain size, orientation and surface structure of the electrodes changed significantly with the deposition temperature. For all electrodes, increase in deposition temperature improved the photocurrent density. Additional amplification of deposition temperature could not carry on after a definite point owing to the volatility of the glass substrate at an elevated temperature (>500 °C), premature decomposition of aerosol and film adherence issues experienced at high temperatures. Therefore, the surface structure and specific orientation are the vital parameters for estimating the wavelength and charge transfer properties [41].

**Figure 6:**
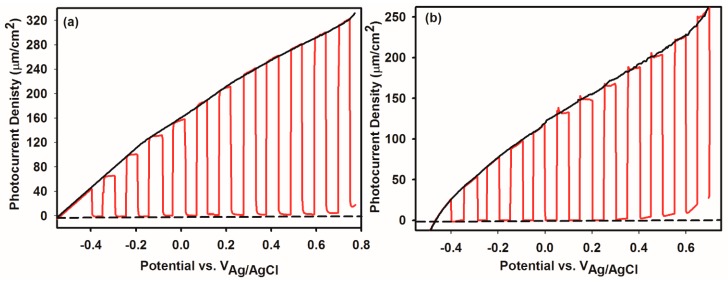
Current–voltage characteristics (**a**) for Hierarchical buckyball-shaped microsphere structured and (**b**) for nanoplates structured for Bi_2_WO_6_ electrodes deposited by AACVD. The light was physically chopped to reveal light and dark current concurrently.

Figure 6 demonstrates the photocurrent measurements under chopping light at regular intervals to reveal light and dark current simultaneously. The steady-state *J-V* plot was overlaid on the chopped plot in each case to compare steady-state and transient photocurrent and both show good agreement. By far the best Bi_2_WO_6_ photoelectrode is that with hierarchical buckyball-shaped microsphere morphology. We attribute this to the hierarchical microsphere structures and (001) preferred orientation plane. The negative shift of the photocurrent onset related to preferred orientation along (001) plane provides a route to engineering the energetic and interfacial charge transfer properties at Bi_2_WO_6_ electrode/electrolyte interface. Along with the preferred crystallographic orientation, the hierarchical microsphere structures have numerous microspores and a high internal surface area that permits more electrolytes in the form of electrolytic solution to enter all microspheres giving a big semiconductor/electrolyte interface. The 220 μAcm^−2^ photocurrent density at 0.23 V (vs. Ag/AgCl/3M KCl) under AM1.5 illumination, as well as a negligible dark current (Figure 6) for hierarchical buckyball-shaped microsphere electrodes. is among the highest photocurrent density for Bi_2_WO_6_ electrodes reported to date. It was revealed that the light absorption properties of Bi_2_WO_6_ electrodes influenced by the preparation method will affect the surface structure and specific orientation of the electrodes [42].

### 3.7. Photocatalytic dye Degradation Studies

**Figure 7:**
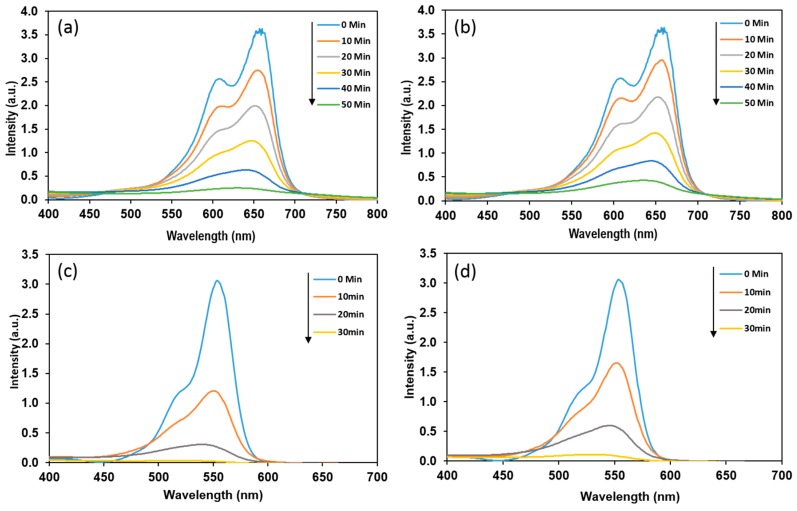
Photocatalytic degradation activity of Bi_2_WO_6_ prepared at different flow rates, (**a**) methylene blue using Bi_2_WO_6_ nanoplates electrodes (MB-P), (**b**) methylene blue using Bi_2_WO_6_ hierarchical buckyball-shaped microsphere electrodes (MB-B), (**c**) rhodamine B using Bi_2_WO_6_ hierarchical buckyball-shaped microsphere electrodes (RhB-B), (**d**) rhodamine B using Bi_2_WO_6_ nanoplates electrodes (RhB-P).

The photocatalytic activity of Bi_2_WO_6_ photoelectrodes was investigated for the decomposition of methylene blue (MB) and rhodamine B (RhB), in visible light illumination. Figure 7 shows the absorbance of MB and RhB dyes at different intervals during the photocatalytic reaction. Electrodes with a hierarchical buckyball-shaped microsphere structure exhibit higher photocatalytic activity than electrodes with nanoplates structures. The Bi_2_WO_6_ photoelectrodes show a much higher photocatalytic efficiency for RhB dye compared to MB. After 30 min of visible light illumination, the RhB removal over Bi_2_WO_6_ photoelectrodes with a hierarchical buckyball-shaped microsphere structure arrives at 99%, obviously higher than the value of 65% for the MB dye. Meanwhile, Bi_2_WO_6_ photoelectrodes with nanoplates structures show photocatalytic efficiency of 90% and 60% for RhB and MB respectively, after the same duration of light illumination.

**Figure 8:**
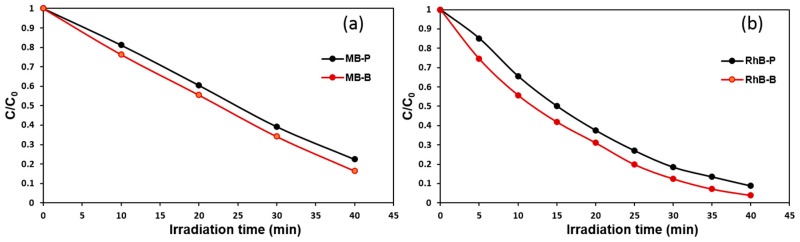
The plot of C/C_0_ vs. time of irradiation for Bi_2_WO_6_ films (**a**) for MB dye and (**b**) for RhB dye.

The photocatalytic efficiency of Bi_2_WO_6_ photoelectrodes in the photochemical decomposition of MB and RhB was determined using the following expression:

(1) $$\%\;Degradation=\frac{C_{0}-C}{C_{0}}\times 100$$

where $C_{0}$ is the initial concentration of dye in mg/L and *C* is the remaining concentration of dye after illumination in time interval. The efficiency of the photocatalysis of Bi_2_WO_6_ photoelectrodes was estimated by photodegradation of MB and RhB as a function of $C_{0}/C$ versus duration of irradiation as shown in Figure 8.

**Figure 9:**
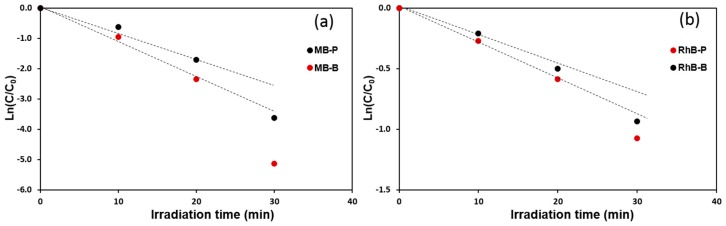
Kinetic plot of Ln(C/C_0_) vs. time of irradiation for photocatalytic degradation of (**a**) MB dye and (**b**) with RhB dye.

The initial decomposition reaction was calculated using pseudo-first-order kinetic law.

(2) $$-\mathrm{Ln}\frac{C}{C_{0}}=k_{app}t$$

where $k_{app}$ is the pseudo-first-order rate constant and t is the time of reaction. In order to confirm the estimation, Ln(C/C_0_) versus illumination time yields a straight line with a slope of $k_{app}$ as shown in Figure 9. Because the straight lines were obtained as expected, the kinetics of RhB photo-degradation described the first-order degradation curve, which coincides with the L-H model [43].

The reaction rate constants ($k_{app}$) observed for photodegradation are pseudo-first-order kinetics and the half-life parameters are presented in Table 1. The data present in the Table 1 approve the observation described previously, where the decrease in the flow rate leads to the formation of a hierarchical buckyball-shaped microsphere structured electrode, which exhibits a photocatalytic activity higher than a nanoplates structured electrode. The observed increase in rate constants of Bi_2_WO_6_ electrodes with a hierarchical buckyball-shaped microsphere structure could be attributed to their morphological and crystalline properties [44]. SEM of Bi_2_WO_6_ electrodes with a hierarchical buckyball-shaped microsphere structure presents greater crystallinity and particle size in comparison with the nanoplates structured electrode, and considering that the higher crystallinity of a material shows lower defects on its surface, which generally act as electron-hole recombination centres; thus, materials with high crystallinity possess high photocatalytic activity [45,46]. In Table 1, it can be observed that the removal rate of RhB dye was better than that of MB dye. After 30 min of exposure to visible light, the RhB removal over Bi_2_WO_6_ electrodes with a hierarchical buckyball-shaped microsphere structure was 99%, obviously higher than the value of 65% for MB, as shown in Figure 7. This could be attributed to the variation in dye chemical structure, which leads to variance in adsorption properties and in capability to degradation [47].

## 4. Conclusions

We have demonstrated, for the first time, a simple method for the preparation of Bi_2_WO_6_ electrodes with nanoplates and hierarchical buckyball-shaped microsphere structured morphologies by appropriately controlling the parameters of the AACVD process. The fabrication method, precursor solution, deposition temperature and carrier gas flow have a big impact on the surface structure and orientation of Bi_2_WO_6_ electrodes. Electrodes can be reproduced easily and show a bandgap energy of ~2.8 eV and display anodic photocurrent. Bi_2_WO_6_ electrodes with a hierarchical buckyball-shaped microsphere structure have shown a photocurrent density of 220 μAcm^−2^ while nanoplates had 170 μAcm^−2^ photocurrent density at 0.23 V (vs. Ag/AgCl/3M KCl) in AM1.5 illumination. Similarly, Bi_2_WO_6_ electrodes with a hierarchical buckyball-shaped microsphere structure have 99% RhB and 65% of MB photocatalytic degradation in 30 min while nanoplates showed photocatalytic efficiency of 90% and 60% for RhB and MB, respectively.

In this work, we have shown that PEC and photocatalytic properties of Bi_2_WO_6_ electrodes can be significantly enhanced by the systematic control of morphology and orientation. We assume that this study will initiate the utilization of Bi_2_WO_6_ electrodes in diverse fields like photodegradation, electronic and electrochemical applications.

## Figures and Tables

**Table 1:** Pseudo-first order rate constants and half-life for the degradation of MB and RhB dyes.

Catalysts	$k_{app}(\min^{-1})$		*t*_1/2_ (min)	
	MB	RhB	MB	RhB
**Bi_2_WO_6_-B**	0.054	0.157	20	7
**Bi_2_WO_6_-P**	0.044	0.116	23	12

## Supplementary Materials

The following are available online at https://www.mdpi.com/2079-4991/9/5/755/s1, Figure S1: Raman Spectra of Bi_2_WO_6_ electrodes, Figure S2: The SEM micrograph of Bi_2_WO_6_ electrode deposited bt AACVD using Bi(NO_3_)_3_·5H_2_O and (NH_4_)_6_ H_2_W_12_O_40_·XH_2_O solution in distilled water without dimethylaminopropanol (DMAP) deposited at 500 °C.
