# Supplementary material for: Highly Efficient Nanostructured Bi2WO6 Thin Film Electrodes for Photoelectrochemical and Environment Remediation
Source: Nanomaterials (Basel). 2019 May 17;9(5):755. doi: 10.3390/nano9050755 (PMC6566810; doi:10.3390/nano9050755)
Supplement: Supplementary file 1 [file nanomaterials-09-00755-s001.pdf]

Highly Efficient Nanostructured Bi_2WO_6 Thin Film Electrodes for Photoelectrochemical and Environment Remediation

Bandar Y. Alfaifi [1], Hossein Bayahia [2] and Asif Ali. Tahir [1,*]

1 Environment and Sustainability Institute (ESI), University of Exeter, Penryn Campus, Penryn, Cornwall TR10 9FE, UK; ba283@exeter.ac.uk
2 Chemistry Department, Faculty of Science, Albaha University, Albaha 65527, Saudi Arabia; hbayahia@bu.edu.sa
* Correspondence: a.tahir@exeter.ac.uk

Supplementary Data

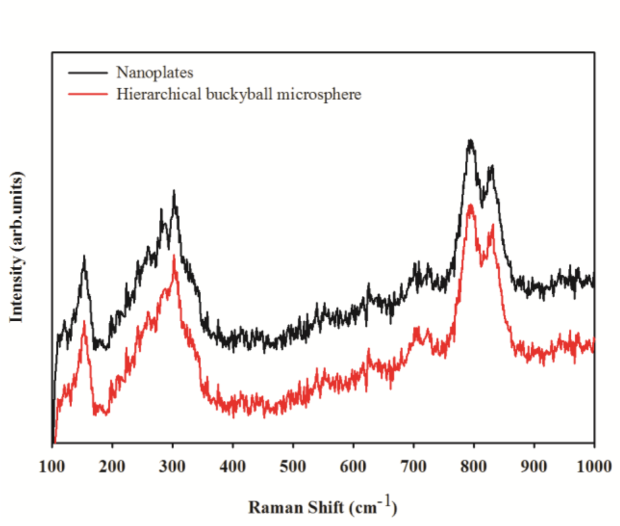

Figure S1: Raman Spectra of Bi_2WO_6 electrodes.

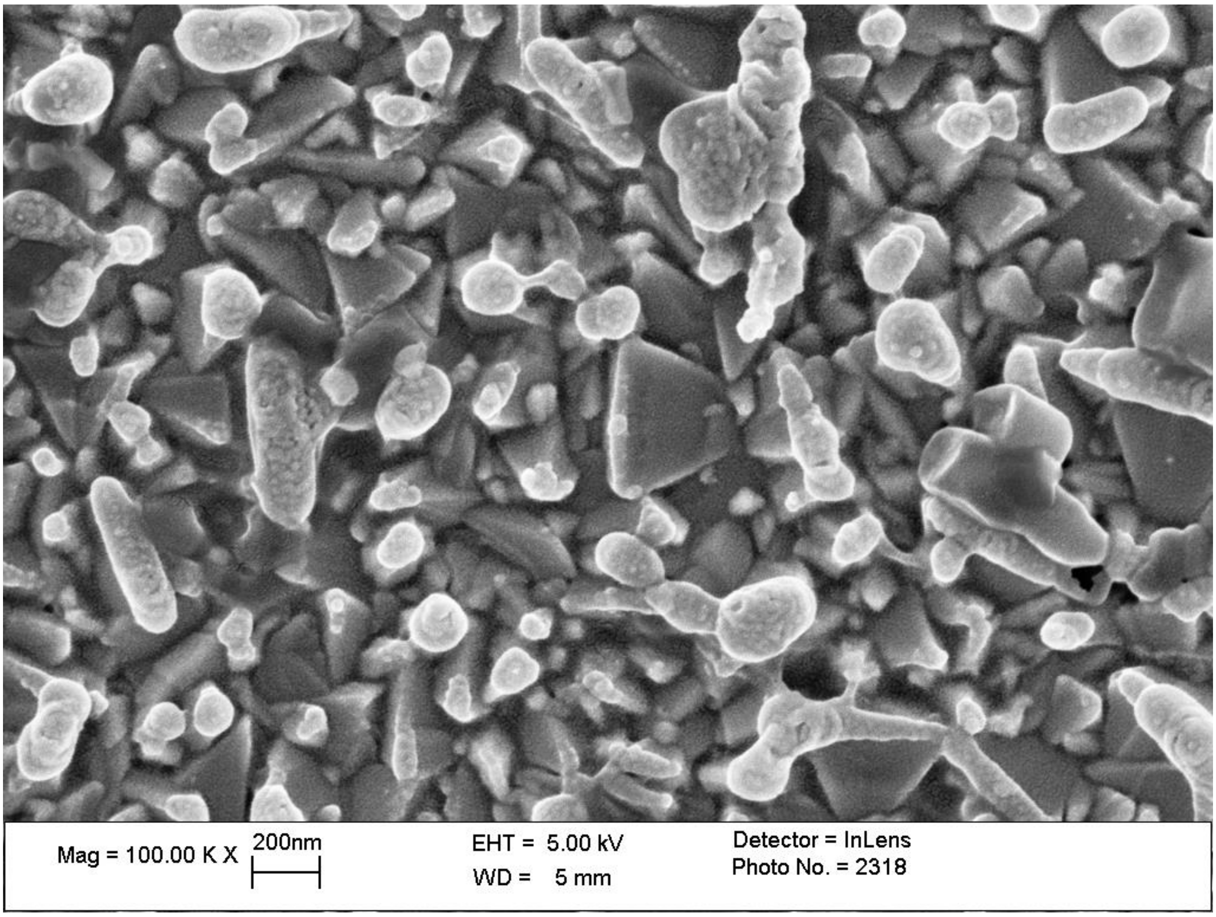

Figure S2: The SEM micrograph of Bi_2WO_6 electrodes deposited by AACVD using aqueous solution of $Bi(NO_3)_3{\cdot}5H_2O$ and $(NH_4)_6\ H_2W_{12}O_{40}{\cdot}XH_2O$ at 500 °C.
